# Mechanoreception for Soft Robots via Intuitive Body Cues

**DOI:** 10.1089/soro.2018.0135

**Published:** 2020-04-03

**Authors:** Liangliang Wang, Zheng Wang

**Affiliations:** ^1^ Department of Mechanical Engineering, The University of Hong Kong, Hong Kong, China.; ^2^ Department of Mechanical and Energy Engineering, Southern University of Science and Technology, Shenzhen, China.

**Keywords:** soft robot, mechanoreception, linear pneumatic actuator, body deformation, soft-rigid hybrid gripper

## Abstract

Mechanoreception, the ability of robots to detect mechanical stimuli from the internal and external environments, contributes significantly to improving safety and task performance during the operation of robots in unstructured environments. Various approaches have been proposed to endow robot systems with mechanoreception. In the case of soft robots, the state-of-the-art mechanosensory solutions typically embedded dedicated deformable sensors into the soft body, giving rise to fabrication complexity and signal sophistication. In this study, we propose a novel mechanoreception scheme to enable pneumatic-driven soft robots to perceive proprioceptive movements as well as external contacts. Both internal and external mechanical parameters can be decoded from intuitive cues of body deformation and pneumatic pressure signals. In contrast to most existing solutions employing dedicated deformable sensors, the proposed approach only utilizes pressure feedback, which is typically available from the pneumatic pressure sensors incorporated in the control loop of most pneumatic soft robots. The concept was implemented and validated on a proprietary robotic gripper with a linear soft pneumatic actuator, demonstrating the capability in simultaneous detection of actuator position and external contact forceAfter the proposed approach, the gripper can achieve both active and passive mechanosensation, with demonstrated experiments in grasping force estimation, contact loss detection, object stiffness identification, and contour measurements. This approach offers an alternative route to achieving excellent internal/environmental awareness without requiring dedicated sensing modalities.

## Introduction

Mechanoreception is a common endowment of humans, which enables the central nervous system with the awareness of any mechanical distortion of the body and the environment in contact.^1^ Various types of mechanoreceptors located within the human body can detect different mechanical stimuli: (1) touch-sensitive cutaneous mechanoreceptors are primarily responsible for reconstructing the size, surface texture, and other tactile features of an object; (2) force-sensitive mechanoreceptors located beneath the skin and inside the body can detect heavy contacts, forces, and the movements of the body segments.^2^ The sensory feedback from mechanoreceptors is critical for humans in achieving proper balance and motor control, dexterous manipulation, and other physical interactions. Without mechanosensory feedback, our physical capabilities would be severely hindered.^3,4^ Similarly for robots, the performances of robot systems will be substantially restrained without sufficient mechanoreceptive information, especially when the robots are operating in an unstructured environment, handling complex tasks, or interacting with humans.^5^

For the past decades, a myriad of approaches have been explored for rigid-bodied robots, achieving both exteroception and proprioception capabilities.^6–9^ However, it remains critically challenging to construct soft robots with mechanoreception since the schemes for rigid robots are untenable due to the distinctive morphology and substantial compliance of soft robots.^10–16^ Recent efforts have been made to integrate various sensing modalities into soft actuators, with examples, including using conductive liquids to achieve proprioceptive soft actuators,^17^ integrating optical fiber sensors into continuum robots for curvature and force sensing,^18^ using customized magnetic curvature sensors on bidirectional bending actuators,^19^ and molding customized strain gauge onto a bending body.^20^ Most recently, soft robots with both proprioception and exteroceptive contact sensing have been reported: a fiber-reinforced soft prosthetic hand employing stretchable optical waveguides was proposed, with the capabilities in detecting shape and texture, probing stiffness, and recognizing objects^21^; a soft somatosensory actuator filled with conductive ionogel and fugitive inks could achieve synergistic curvature, inflation, and contact sensing.^22^ There are also several other sensory solutions proposed to enable soft robots with mechanoreceptive feedback.^23–27^

To detect and distinguish different mechanical cues (e.g., body space movements and external contact force), multiple types of embedded sensors are typically required in the state-of-the-art solutions, leading to structural complexity and fabrication challenges. This study explores a new perspective on mechanoreception. A novel scheme is proposed to simultaneously detect and distinguish the mechanical cues within the soft body or from the external environment by utilizing intuitive body cues and the sensory feedback readily available. For the widely adopted pneumatic elastomeric-bodied soft robots, deformation and interactive force can be obtained simultaneously by the proposed scheme, using the pressure sensors only in the pneumatic control loop.

The conceptual, analytical, and experimental study on mechanoreception will be presented in the following order: the overall concept of mechanoreception will be discussed first, followed by the analysis, design, and modeling of a one degree of freedom (1-DoF) linear soft pneumatic actuator constructed with antagonistic pair of bellows chambers. Built on the 1-DoF actuator model, a proprietary soft-robotic gripper will be then proposed, with proprioceptive position sensing and dynamic exteroceptive contact force estimation being formulated. With a fabricated gripper prototype, complete experimental results will be presented, including the calibration tests, gripper evaluation experiments, and the mechanoreception experiments, followed by conclusions and future study.

## Concept of the Proposed Mechanoreception Scheme

A novel scheme on mechanoreception was proposed for a large family of pneumatic soft robots with elastomeric chambers and built-in pneumatic control. The proposed concept was conceived from two aspects:

(1) Gas pressure sensors already embedded in the pneumatic control loop can be used to detect the body deformation, which can then be used to reveal the actuated movement and external mechanical stimuli.
(2) Prior models of the static and dynamic behavior of body deformation can be used to decode the proprioceptive and exteroceptive parameters from the measured pressure feedback.

**FIG. 1.**
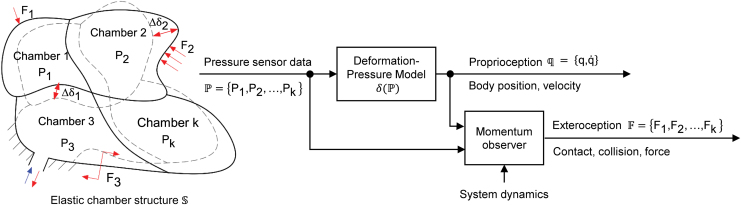
Mechanoreception concept for soft bodied system. Color images are available online.

The concept of the proposed mechanoreception scheme for soft-bodied systems is summarized in Figure 1. The patterned deformable chambers delicately constructing the soft body were designed to serve as actuators as well as mechanoreceptors. Both actuated movements and external mechanical stimuli can be signaled by the inner pressure change of the deformable chambers. The proprioceptive states such as actuator position and velocity can be obtained by modeling the relationship between the body movements and the changes in chamber pressure. With the status of body movements being learned, a momentum observer could be further introduced to decode external forces.

Notably, compared with the state-of-the-art solutions with dedicated sensors, the proposed method only uses readily available pressure sensors. The practicality of external mechanical sensing based on pressure sensors has been already explored, including the commercialized biomimetic tactile sensor BioTac^®^ (SynTouch LLC) that adopted the pressure sensors to detect slip-related microvibrations,^28,29^ tactile array sensor customized by inexpensive barometric pressure sensors,^30^ and soft sensors composed of a sensing body and a pressure sensor for contact force and object curvature measurements.^31^ However, these ideas did not tackle the problem of proprioception or the situation that body movements coexist with external mechanical stimuli, which is the aim of the mechanoreception scheme proposed in this study.

## Design and Characterization of a 1-DoF Soft Pneumatic Bellows Actuator

To endow pneumatic soft robots with mechanoreception, the prominent challenge is that the actuated pneumatic pressure and the resultant pressure from environmental interaction are coupled. In this section, a 1-DoF soft actuator is proposed with antagonistically configured dual pneumatic chambers, each embedded with a pressure sensor. The two simultaneous but independent pressure feedback can provide sufficient information to distinguish between the internal status and external stimuli.

### Antagonistic design of a soft actuator with bellows structure

**FIG. 2.**
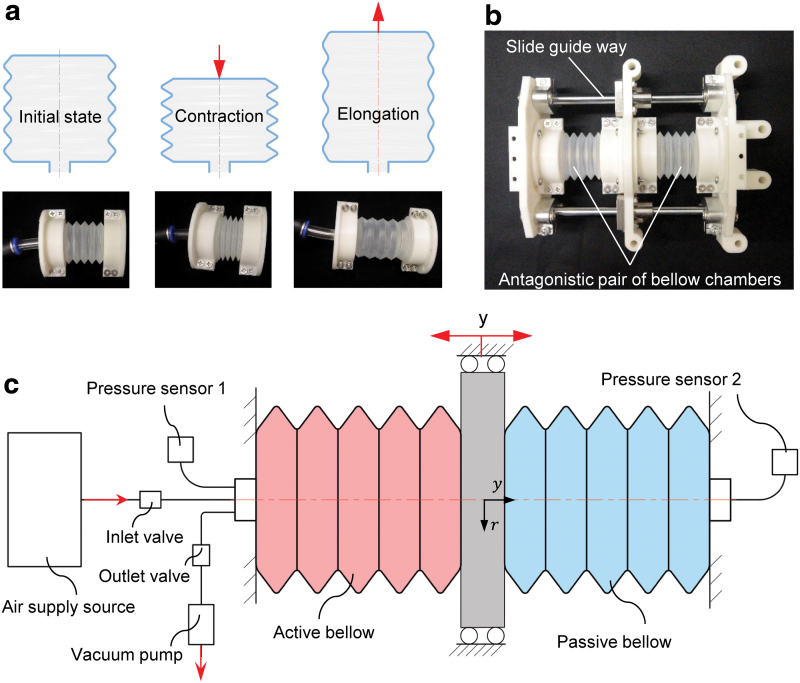
Design of the proposed antagonistic bellow actuator: **(a)** Deformation mode of the bellow chambers. The V-shape circle-round-type bellow concentrates elongation or contraction in axial direction. **(b)** Mechanical structure of the proposed antagonistic bellow actuator. **(c)** Schematic representation of the antagonistic bellow actuator. Color images are available online.

In terms of the structure, we constructed the soft actuator with two antagonistic V-shape circle-round-type bellows chambers.^32–37^ Among inflated soft robotic actuators, bellows chambers have been widely employed for their simplicity and sensitivity to single-DoF deformation.^38–43^ The bellows chambers used in this study concentrate elongation/contraction motion in the axial direction. Figure 2a shows the overall deformation of a bellows chamber.

For simplicity of derivation without losing generality, a 1-DoF linear soft pneumatic actuator with dual bellows chambers was proposed to verify the proposed concept. The bellows chambers were constrained antagonistically along the axial direction as shown in Figure 2b and the operating principle is illustrated in Figure 2c. In the proposed dual chambers design, an active bellows chamber was connected to a pressure sensor and the air supply line, whereas a passive bellows chamber was connected to a second pressure sensor only. By regulating the supplied pressure to the active bellows chamber, the position of a slider connected to the two chambers can be controlled. The inner pressure of the passive bellows chamber accounted for the length and movement velocity of the bellows chamber and, therefore, played a vital role in proprioception. External mechanical stimuli along the axial direction can be obtained by using the inner pressures of the active and passive bellows chamber synergistically.

### Analytical modeling of bellows chambers

The mechanical property of the bellows chamber is of critical significance as it indicates how mechanical cues are encoded by the proposed structure into pressure signals. The deformable bellows chamber could both be actuated or react to external mechanical stimuli; thus, the inherent stiffness of the bellows structure is a fundamental quantity in decoupling them. Therefore, the axial stiffness of the bellows chamber was modeled to reveal the ingenuity of the antagonistic structure for addressing the nonlinearity problem.

The deformable bellows chamber used in this study was fabricated with low-density polyethylene (LDPE) by blow molding. The structural and material parameters of the LDPE bellows are presented in Table 1.

**Table 1.** Structure and Material Parameters of the Bellow Chamber

Structure parameters	
Outer diameter (mm)	*D*
Inner diameter (mm)	*d*
Initial length of one convolution (mm)	*h*_0_
Initial outer diameter (mm)	*D*_0_
Outer diameter/inner diameter ratio	α=D∕d
Wall thickness (mm)	*t*
Number of convolutions	*N*

Material parameters (low-density polyethylene)	
Modulus of elasticity E (MPa)	172
Poisson's ratio μ	0.439

**FIG. 3.**
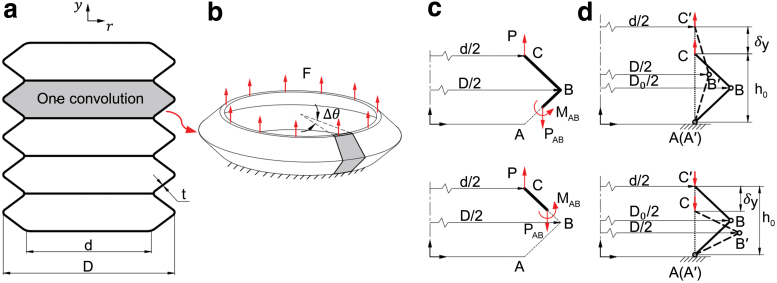
Axial stiffness analysis of the bellow structure. **(a)** Structure parameters of the bellow structure. **(b)** Force and boundary condition of one convolution of bellow. **(c)** Static analysis of a sector *Δθ* of one convolution. **(d)** Simplified geometry model for analyzing the relationship between the outer diameter and inner diameter ratio *α* and displacement *y*. Color images are available online.

Considering that all V-shape convolutions of the bellows are identical, as shown in Figure 3a, we first consider the relationship between the axial load and the deflection of one V-shape convolution. In this case, the inner circular edge of one end is clamped and a dummy axial load *F* is introduced, distributing uniformly around the circumference of the other end, as illustrated in Figure 3b. Consider a sector Δθ of one convolution, the axial deflection $\delta_{y}$ at point C could be deduced by the Castigliano's theorem,^44^

(1) $$\delta_{y}={\left(\frac{\partial U}{\partial P}\right)}_{AB}+{\left(\frac{\partial U}{\partial P}\right)}_{BC}=\int_{OA}\frac{M_{AB}}{EI_{AB}}\frac{\partial M_{AB}}{\partial P}+\int_{AB}\frac{M_{BC}}{EI_{BC}}\frac{\partial M_{BC}}{\partial P}.$$

From the static analysis shown in Figure 3c,

$$\begin{array}{c} P_{AB}=P_{BC}=P=F\frac{\Delta \theta }{2\pi },M_{OA}=M_{AB}=P\left(r-\frac{d}{2}\right),\\ I_{OA}=I_{AB}=\frac{t^{3}}{12\left(1-\mu^{2}\right)}r\Delta \theta . \end{array}$$

Hence

(2) $$\delta_{y}=2\int_{\frac{d}{2}}^{\frac{D}{2}}\frac{F\frac{\Delta \theta }{2\pi }\left(r-\frac{d}{2}\right)}{\frac{Et^{3}}{12\left(1-\mu^{2}\right)}r\Delta \theta }\left(r-\frac{d}{2}\right)dr.$$

Solving the integral equation in Equation (2), the axial deflection $\delta_{y}$ of one convolution under axial load *F* could be obtained as

(3) $$\delta_{y}=\frac{3\left(1-\mu^{2}\right)d^{2}}{\pi Et^{3}}\left[\ln\alpha -\left(\alpha -1\right)+\frac{{\left(\alpha -1\right)}^{2}}{2}\right]F,$$

where α=D∕d.

Multiplying by *N* gives the total axial displacement *y* for a bellows with *N* convolution under axial load *F*,

(4) $$y=N\delta_{y}=\frac{3N\left(1-\mu^{2}\right)d^{2}}{\pi Et^{3}}\left[\ln\alpha -\left(\alpha -1\right)+\frac{{\left(\alpha -1\right)}^{2}}{2}\right]F.$$

Note that the displacement of the bellows is defined as positive when elongated and negative when compressed.

From Equation (4), the axial stiffness *k_y_* could be written as

(5) $$k_{y}=\frac{F}{y}=\frac{\pi Et^{3}}{3N\left(1-\mu^{2}\right)d^{2}\left[\ln\alpha -\left(\alpha -1\right)+\frac{{\left(\alpha -1\right)}^{2}}{2}\right]}.$$

The axial stiffness of the bellows is a function of four structural parameters, *N*, *d*, *t*, α, among which *N* and *t* can be regarded as fixed-structure parameters during deformation. As the inner circular edge is clamped, the inner diameter of the bellows is assumed to remain unchanged, whereas the outer diameter of the bellows is of free variation during deformation. Thus, the outer diameter and inner diameter ratio α of the bellows varies during elongation or contraction, suggesting that the axial stiffness of the bellows can change with deformation. A simplified geometrical model is proposed for analyzing the relationship between α and displacement *y*, as shown in Figure 3d, where the V-shape convolution is simplified as a two-bar linkage. The free end (point *C*) of the linkage is assumed to move along the axial direction. The initial length and initial outer diameter of the V-shape convolution are *h*_0_ and *D*_0_, respectively.

From the simplified geometry model, we have

(6) $${\left(\frac{h_{0}}{2}\right)}^{2}+{\left(\frac{D_{0}}{2}-\frac{d}{2}\right)}^{2}={\left(\frac{h_{0}+\delta_{y}}{2}\right)}^{2}+{\left(\frac{D}{2}-\frac{d}{2}\right)}^{2}.$$

Solving Equation (6), the relationship between the ratio α and axial displacement *y* could be derived as

(7) $$\alpha \left(y\right)=\frac{D}{d}=1+\frac{\sqrt{h_{0}^{2}+{\left(D_{0}-d\right)}^{2}-{\left(\frac{y}{N}+h_{0}\right)}^{2}}}{d}.$$

Consider the following two boundary conditions:

(1) The V-shape bellows chamber is elongated to a cylindrical shell with diameter *d*. In this case, the bellows chamber is mainly under tensile deformation rather than bending deformation. The axial stiffness becomes steeply large.
(2) The V-shape bellows chamber is compressed to the situation that the adjacent convolution adheres to each other. The axial stiffness exhibits a sudden surge as it switches from bending deformation to compression deformation.

The bellows chamber can be deformed between these two boundaries. By calculating the displacement *y* of the two boundary conditions, the workable displacement range can be obtained as

(8) $$-N\left(h_{0}-2t\right)<y<N\left(\frac{\sqrt{h_{0}^{2}+{\left(D_{0}-d\right)}^{2}}}{2}-h_{0}\right).$$

Thus, within the workable displacement range, the outer diameter and inner diameter ratio α meets

(9) $$1<\alpha \left(y\right)<1+\frac{\sqrt{h_{0}^{2}+{\left(D_{0}-d\right)}^{2}-4t^{2}}}{d}.$$

**FIG. 4.**
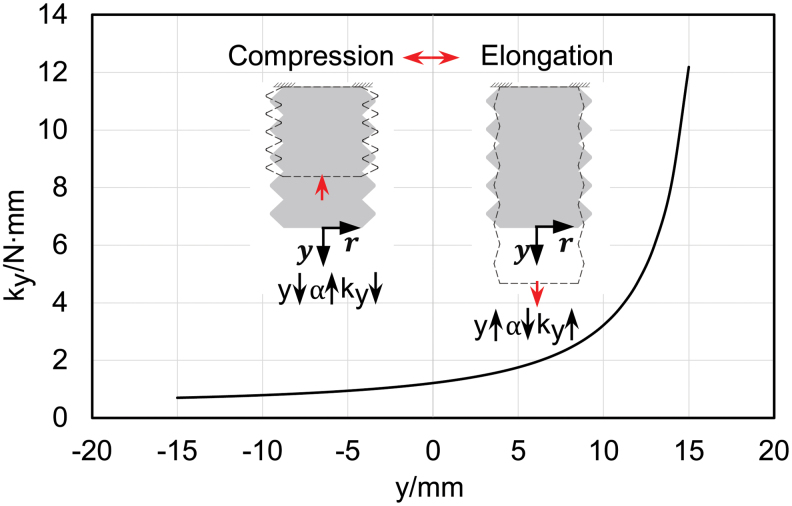
Nonlinear trend of the axial stiffness for bellow structure with parameters *d* = 25 mm, *t* = 0.5 mm, *N* = 5, *D*_0_ = 32 mm, and *h*_0_ = 6 mm. Color images are available online.

Equation (7) shows that in the workable displacement range, the outer diameter and inner diameter ratio α decreases as the axial displacement *y* increases. From Equation (5), the axial stiffness *k_y_* is a monotonic decreasing function of the outer/inner diameter ratio α. Therefore, we can conclude that the axial stiffness of the bellows structure is nonlinear and it grows exponentially with the axial displacement increasing. Figure 4 presents the nonlinear trend of the axial stiffness for a bellows chamber with parameters: *d* = 25 mm, *t* = 0.5 mm, *N* = 5, *D*_0_ = 32 mm, *h*_0_ = 6 mm.

The nonlinear characteristics of the axial stiffness of the bellows structure will cause difficulties in further modeling. However, the introduction of dual antagonistic bellows contributes to the linearization. By exploiting the structural symmetry, the axial stiffness of the antagonistic actuator denoted as *k_a_* can be obtained as


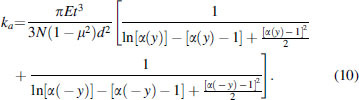


The workable displacement of the antagonistic bellows actuator becomes

(11) $$\left|y\right|<\min\left\{N\left(h_{0}-2t\right),N\left[\frac{\sqrt{h_{0}^{2}+{\left(D_{0}-d\right)}^{2}}}{2}-h_{0}\right]\right\}.$$

**FIG. 5.**
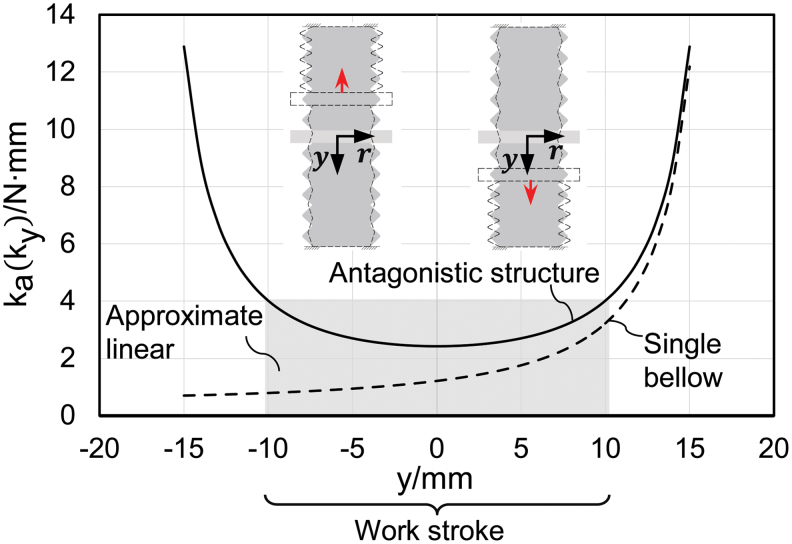
Approximately linear trend of the axial stiffness for antagonistic bellow structure in work stoke (*solid curve*) compared with the nonlinear trend of the axial stiffness for single bellow structure (*dotted curve*) with parameters *d* = 25 mm, *t* = 0.5 mm, *N* = 5, *D*_0_ = 32 mm, and *h*_0_ = 6 mm. Color images are available online.

For the antagonistic structure, when one bellows is being elongated with increasing stiffness, the other one is being compressed for the same axial displacement with decreasing stiffness. Hence, the overall effect of deformation on actuator stiffness is reduced by the proposed antagonistic design. As shown in Figure 5, the work stroke of the antagonistic bellows actuator corresponds to the middle interval of the workable displacement range, thereby achieving approximated linear stiffness. The analytic model of the antagonistic structure is substantially simplified because of the reduced nonlinearity. The desired working stiffness could be obtained by optimizing the structural parameters *N*, *t*, *d*, *D*_0_, and *h*_0_.

By denoting the axial load of the bellows as $F_{b}\left(y\right)$, the relationship between axial load and actuator displacement could then be derived as

(12) $$F_{b}\left(y\right)=\int k\left(y\right)dy=\int \frac{\pi Et^{3}}{3N\left(1-\mu^{2}\right)d^{2}\left[\ln\left[\alpha \left(y\right)\right]-\left[\alpha \left(y\right)-1\right]+\frac{{\left[\alpha \left(y\right)-1\right]}^{2}}{2}\right]}dy.$$

Let g be the nonintegrable function defined by $g\left(\alpha \right)=\frac{1}{\ln\alpha -\left(\alpha -1\right)+\frac{{\left(\alpha -1\right)}^{2}}{2}}$. On substituting the Taylor expansion of $\ln\alpha \left(\ln\alpha =\sum_{k=1}^{\infty }\frac{{\left(-1\right)}^{k-1}{\left(\alpha -1\right)}^{k}}{k}\right)$ into Equation (12), a lower order approximation of g (α) could be obtained as

(13) $$g\left(\alpha \right)=\frac{1}{\frac{{\left(\alpha -1\right)}^{3}}{3}-O\left[{\left(\alpha -1\right)}^{3}\right]}.$$

In practical design, the difference between the outer and inner diameters is small, hence the term $\left(\alpha -1\right)\gg 1$. Thus, substituting the third-order approximation of Taylor expansion of$g\left(\alpha \right)$ into Equation (5) yields


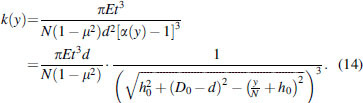


Substituting Equation (14) into Equation (12), the relation between the axial load $F_{b}\left(y\right)$ and the displacement *y* for a single bellows unit could be obtained as

(15) $$F_{b}\left(y\right)=\frac{\pi Et^{3}d}{\left(1-\mu^{2}\right)\left[h_{0}^{2}+{\left(D_{0}-d\right)}^{2}\right]}\cdot \left(\frac{\frac{y}{N}+h_{0}}{\sqrt{h_{0}^{2}+{\left(D_{0}-d\right)}^{2}-{\left(\frac{y}{N}+h_{0}\right)}^{2}}}-\frac{h_{0}}{D_{0}-d}\right).$$

Similarly, we could obtain the relation between the axial load $F_{b}\left(y\right)$ and the displacement *y* for an antagonistic bellows structure

(16) $$F_{a}\left(y\right)=\frac{\pi Et^{3}d}{\left(1-\mu^{2}\right)\left[h_{0}^{2}+{\left(D_{0}-d\right)}^{2}\right]}\cdot \left(\frac{\frac{y}{N}+h_{0}}{\sqrt{h_{0}^{2}+{\left(D_{0}-d\right)}^{2}-{\left(\frac{y}{N}+h_{0}\right)}^{2}}}+\frac{-\frac{y}{N}+h_{0}}{\sqrt{h_{0}^{2}+{\left(D_{0}-d\right)}^{2}-{\left(-\frac{y}{N}+h_{0}\right)}^{2}}}-\frac{2h_{0}}{D_{0}-d}\right).$$

**FIG. 6.**
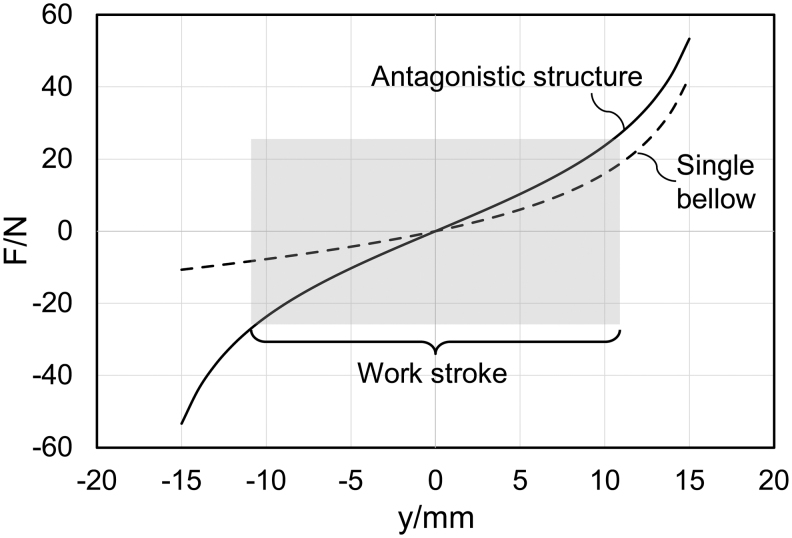
The axial load and displacement characteristics of single bellow structure (*dashed curve*) and antagonistic bellow structure (*solid curve*) with parameters *d* = 25 mm, *t* = 0.5 mm, *N* = 5, *D*_0_ = 32 mm, and *h*_0_ = 6 mm.

Figure 6 illustrates the general trend between the axial load and displacement characteristics of a single bellows structure and the antagonistic bellows structure with parameters *d* = 25 mm, *t* = 0.5 mm, *N* = 5, *D*_0_ = 32 mm, and *h*_0_ = 6 mm.

## Mechanoreception Scheme

The antagonistic bellows actuator consisted of one active bellows chamber connecting to the air supply source for active pneumatic regulations, and a passive one that was sealed for pressure monitoring only. By inflating or deflating the active bellows chamber, the pressure differential between the active bellows chamber and passive bellows chamber could drive the slider connecting the two bellows chambers along the guide. Accordingly, the pressure changes in the sealed passive bellows chamber could provide feedback on the actuator deformation. External mechanical stimuli acting on the slider would also induce axial compression or elongation of the two bellows chambers. Pressure changes in the two bellows chambers can be observed and varied under both internal and external mechanical stimuli. To distinguish the internal and external stimuli, the proprioceptive sense of actuator displacement can be achieved first from the pressure measurements of the sealed passive bellows chamber. With the status of actuator deformations being obtained, the external force can then be decoded by incorporating system dynamics.

### Proprioception in actuator displacement

For a sealed chamber with an ideal gas, pressure and temperature within the chamber are assumed to be uniformly distributed, and the kinetic and potential energy of the air is negligible. Based on Boyle's law, we can obtain

(17) PV=k,

where *P* is the pressure of the gas, *V* is the volume of the gas, and *k* is a constant.

From Boyle's law, the volume is inversely proportional to internal pressure for a fixed amount of gas kept at a fixed temperature. Thus, it can be used to predict the changes in volume by measuring the changes in pressure. For a sealed bellows chamber with one end fixed and the other end deforms along the axial direction, the volume of the bellows chamber can be obtained based on the simplified geometry model proposed in the last section:

(18) $$V=V_{inactive}+\frac{1}{12}\pi \left(Nh_{0}-y\right)\left(d^{2}+D^{2}+dD\right),$$

where $V_{inactive}$ is the inactive volume at the end of the bellows chamber, including the volume of the tube connecting the bellows chamber to the pressure sensor. With the bellows deforming within the workable displacement range, the outer and inner diameters are very close and the change of the outer diameter is small compared with the axial displacement. Thus, the term $\left(d^{2}+D^{2}+dD\right)$ can be taken as a constant and we define$A=\frac{1}{12}\pi \left(d^{2}+{D_{0}}^{2}+dD_{0}\right)$. The normalized inactive length associated with the inactive volume in bellows chamber can then be defined as $S_{inactive}=V_{inactive}∕A$. Thus, the volume of bellows chamber can be expressed as

(19) $$V=A\left(Nh_{0}+S_{inactive}-y\right).$$

Given the initial volume *V*_0_ and pressure $P_{0}$of the sealed bellows chamber at the initial length (y=0), combining Equation (19) and the Boyle's law we could obtain

(20) $$PA\left(S-y\right)=P_{0}AS,S=Nh_{0}+S_{inactive}.$$

Therefore, the axial displacement of a seal bellows chamber can be indicated by its pressure through the following equation:

(21) $$y=\frac{\left(P-P_{0}\right)}{P}S.$$

When it is under the actuating movement only or coexisting with external mechanical stimuli along the axial direction, the actuator displacement can be mapped by the pressure of the passive bellows chamber,

(22) $$y=\frac{\left(P_{2}-P_{0,2}\right)}{P_{2}}S_{2},S_{2}=Nh_{0}+S_{inactive,2}.$$

### Dynamic exteroceptive contact force estimation

To better demonstrate the implementation of mechanical stimuli detection, a two-fingered gripper actuated by the antagonistic bellows actuator is presented in this section. As the end-effector of the robot manipulator, grippers are of fundamental importance in performing physical interactions with the environment. Integrated with the proposed antagonistic bellows actuator, the gripper was endowed with inherent adaptability and compliance. It would be an ideal exemplar to showcase how actuator-level detection of external mechanical stimuli was achieved when the gripper performed unstructured grasping tasks.

**FIG. 7.**
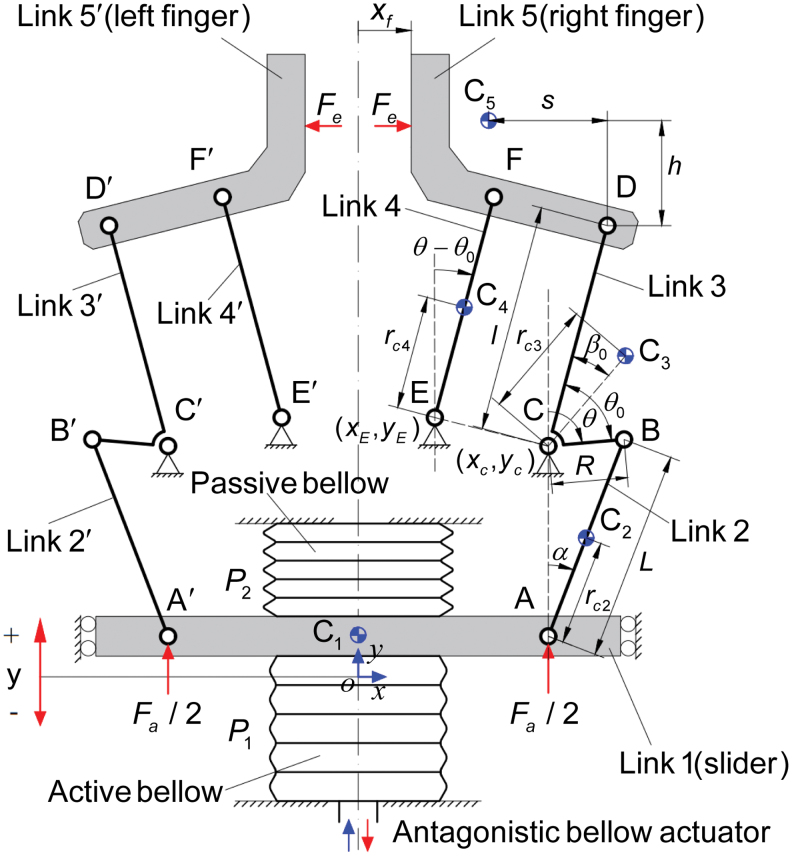
Schematic diagram of gripper system integrated with the antagonistic bellow actuator. Color images are available online.

The mechanism of the proposed soft-rigid hybrid gripper is illustrated in Figure 7. The gripper was constructed with two symmetric 6-bar linkage with link 2 and link 2′ connected to the slider of the antagonistic bellows actuator, enabling synchronous and symmetrical operation of the two fingers (link 5 and link 5′).^45,46^ The single-DoF 6-bar linkage had two closed-loop mechanisms: a slider-crank mechanism for closed-loop 1, and a parallelogram linkage for closed-loop 2. Link 3 (or link 3′) served as the connector of the two closed-loop mechanisms. Actuator displacement *y*, angles α, θ were selected as a set of generalized coordinates. The angles were measured between the positive *y*-axis and links. The nomenclature used for modeling of the gripper system is shown in Table 2.

**Table 2.** Nomenclature of the Gripper System

Nomenclature of the gripper system	
*L*	Distance between joint A and joint B
*R*	Distance between joint C and joint B
*l*	Distance between joint C (joint E) and joint D (joint F)
$r_{c2}$	Distance between the mass center C_2_ of Link 2 and joint A
$r_{c3}$	Distance between the mass center C_3_ of Link 3 and joint C
$r_{c4}$	Distance between the mass center C_4_ of Link 4 and joint E
*s*	Distance between the mass center C_5_ of Link 5 and joint D along *x*-axis direction
*h*	Distance between the mass center C_5_ of Link 5 and joint D along *y*-axis direction
$\left(x_{C},y_{C}\right)$	Coordinate of joint C (fixed)
$\left(x_{E},y_{E}\right)$	Coordinate of joint E (fixed)
$\left(x_{C_{i}},y_{C_{i}}\right)$	Coordinate of the mass center *C_i_*
*y*	Displacement of antagonistic bellow actuator (slider) along *y*-axis direction
α	Angle between vector AB and the positive *y*-axis
θ	Angle between vector CB and the positive *y*-axis (angle position of Link 3)
$\theta_{0}$	Angle between vector CD and vector CB (fixed)
$\beta_{0}$	Angle between vector CD and vector CC_3_ (fixed)
*x_f_*	Position of right fingertip in *x*-axis
*m_i_*	Mass of Link i,i=1,2,…,5
*I_i_*	Moment of inertia of Link *i* about the axis through the mass center perpendicular to o*xy* plane, i=2,3,4
*P*_1_	Pressure of active bellow chamber
*P*_2_	Pressure of passive bellow chamber
*F_a_*	Actuation force of antagonistic bellow actuator induced by the pressure differential
*F_e_*	External force (contact force) acting on Link 5 (Link 5’) along *x*-axis direction

A momentum-based disturbance observer was introduced and extended to the gripper system for external force estimation.^9,47,48^ The observer assumed the presence of force/torque disturbances in the joints, which was considered to be produced by the 1-DoF grasping force at the gripper fingertips. Including the disturbance force, the dynamic model of the gripper system expressed by the generalized coordinate θ takes the following form (complete derivation of the dynamic equation is given in Appendix 1):

(23) $$M\left(\theta \right)\ddot{\theta }+C\left(\theta ,\dot{\theta }\right)\dot{\theta }+g\left(\theta \right)+u\left(\theta \right)=Q_{\theta },$$

where$M\left(\theta \right)$,$C\left(\theta ,\dot{\theta }\right)$,$g\left(\theta \right)$,$u\left(\theta \right)$ and $Q_{\theta }$ are presented in Appendix 1. Particularly,

(24) $$u\left(\theta \right)=k_{a}y\frac{\partial y}{\partial \theta },$$

where *k_a_* is the stiffness of the antagonistic bellows structure, *y* is the displacement of the antagonistic bellows actuator. Here$u\left(\theta \right)$ represents the generalized elastic forces due to the deformation of the antagonistic bellows structure. The generalized force $Q_{\theta }$ corresponding to the generalized coordinate θ is introduced as

(25) $$Q_{\theta }=\tau_{a}\left(P_{1},P_{2},\theta \right)+\tau_{e}\left(\theta \right)=F_{a}\frac{\partial y\left(\theta \right)}{\partial \theta }+F_{e}\frac{\partial x\left(\theta \right)}{\partial \theta },$$

where *F_a_* is the actuation force applied on the slider, which can be estimated by the measured pressure *P*_1_ and *P*_2_ of the active and passive bellows chambers as $F_{a}=\frac{1}{4}\pi d^{2}\left(P_{1}-P_{2}\right)$; *F_e_* is the external contact force applied on the gripper fingertips along *x*-axis, *x* is the displacement of the gripper fingertip along *x*-axis. Friction and other dissipative terms are not considered in the model. $M\left(\theta \right)$ and $C\left(\theta ,\dot{\theta }\right)$ are defined such that

(26) $$Ṁ\left(\theta \right)=\frac{dM\left(\theta \right)}{dt}=2C\left(\theta ,\dot{\theta }\right).$$

According to the dynamic model, the external contact force *F_e_* could have been estimated using position θ, velocity $\dot{\theta }$, and acceleration $\ddot{\theta }$. However, it is not practical to use the second-order differentiation $\ddot{\theta }$ considering the sensory data noise of θ. The proposed momentum observer releases the requirement of acceleration terms by defining a generalized momentum of the gripper system as

(27) $$p=M\left(\theta \right)\dot{\theta }.$$

Taking the first-order derivative over time,

(28) $$p^{.}=M\left(\theta \right)\ddot{\theta }+Ṁ\left(\theta \right)\dot{\theta }.$$

Substituting Equations (23) and (26) into Equation (28) yields

(29) $$\begin{array}{c} p^{\cdot }=F_{a}\frac{\partial y}{\partial \theta }+F_{e}\frac{\partial x}{\partial \theta }-C\left(\theta ,\dot{\theta }\right)\dot{\theta }-g\left(\theta \right)-k_{a}y\frac{\partial y}{\partial \theta }+Ṁ\left(\theta \right)\dot{\theta }\\ =\left(F_{a}-k_{a}y\right)\frac{\partial y}{\partial \theta }+\left(Ṁ\left(\theta \right)-C\left(\theta ,\dot{\theta }\right)\right)\dot{\theta }-g\left(\theta \right)+F_{e}\frac{\partial x}{\partial \theta }\\ =\left(F_{a}-k_{a}y\right)\frac{\partial y}{\partial \theta }+C\left(\theta ,\dot{\theta }\right)\dot{\theta }-g\left(\theta \right)+F_{e}\frac{\partial x}{\partial \theta } \end{array}.$$

Based on the expression of dynamics of *p* and denoting $\hat{p}$ as the prediction of *p*, the observer dynamics can be obtained as

(30) p^⋅=Fa−kay∂y∂θ+Cθ,θ˙θ˙−gθ+r,

where the residual r is defined as$r=K_{O}\left(p-\hat{p}\right)$, and *K_O_* is the observer gain.

The residual dynamics can be obtained as

(31) ṙ=KOṗ−p^⋅=KOFe∂x∂θ−r.

With $R\left(s\right)=L\left\{r\right\}$ and $F\left(s\right)=L\left\{F_{e}\frac{\partial x}{\partial \theta }\right\}$, the residual dynamics in the frequency domain can be produced by taking Laplace transform:

(32) $$R\left(s\right)=\frac{K_{O}}{s+K_{O}}F\left(s\right)=\frac{1}{1+\frac{1}{K_{O}}s}F\left(s\right).$$

**FIG. 8.**
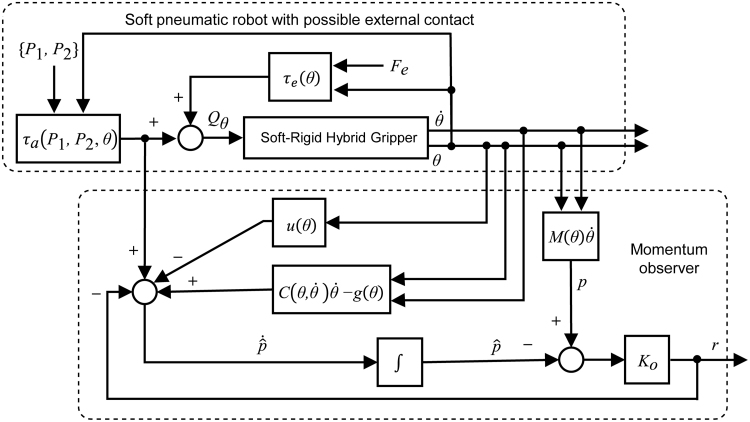
Schematic overview of the momentum observer.

It is observable that the residual *r* would approach the external force by choosing a very large observer gain *K_O_*. Using Equations (29) and (30), the residual *r* can be obtained as


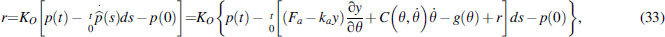


with$p=M\left(\theta \right)\dot{\theta }$. The schematic overview of the proposed momentum observer is illustrated in Figure 8.

Finally, the external grasping force acted on the gripper finger along *x*-axis can be estimated by

(34) $$F_{e}={\left(\frac{\partial x}{\partial \theta }\right)}^{-1}r.$$

## Evaluation Experiment and Calibration

### Force and displacement characteristics of the bellows structure

**FIG. 9.**
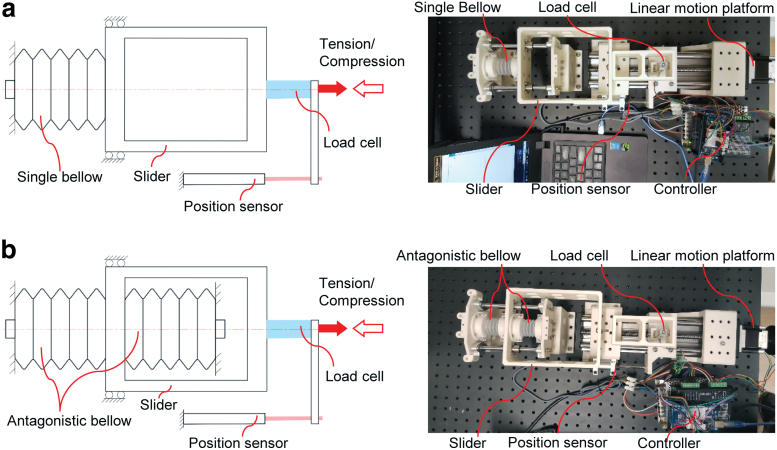
Experimental setup for test of axial force and displacement characteristics of **(a)** single bellow structure and **(b)** antagonistic bellow structure. Color images are available online.

A test apparatus was dedicatedly built to characterize the mechanical property of the bellows structure as shown in Figure 9. According to the boundary constraints in the analytical model, the inner circular edge of the bellows was clamped by a rigid fixture. In the single bellows test, one end of the bellows was fixed, whereas the other end was free by connecting to a slider as illustrated in Figure 9a. In the antagonistic bellows test, the free ends of the two bellows were antagonistically connected to the slider as shown in Figure 9b. A linear motion platform driven by a stepper motor (57BYGH301AA, 200 steps per revolution) was employed to exert tensile or compressive force to the bellows by connecting the guide bearing of the linear motion platform to the slider. A load cell (±100 N max.) was mounted between the slider and the guide bearing of the linear motion platform to measure the axial force applied on the bellows structure. The free rod of a linear position sensor (KPM16, 75 mm max.; MIRAN Ltd.) was connected to the guide bearing of the linear motion platform; thus, the axial displacement of the bellows structure was recorded. The structural parameters of the bellows specimens were *d* = 25 mm, *t* = 0.5 mm, *N* = 5, *D_0_* = 32 mm, and *h_0_* = 6 mm.

**FIG. 10.**
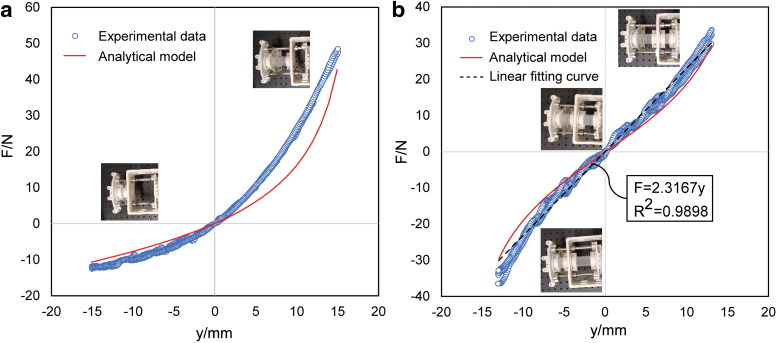
Axial force and displacement characteristics of **(a)** single bellow structure and **(b)** antagonistic bellow structure from experimental results and analytical models. The slope of the *black dotted curve* in **(b)** indicates the measured stiffness of the antagonistic bellow structure used in this study. Color images are available online.

To validate the analytical models, quasi-static tensile/compressive tests of the single bellows structure and the antagonistic bellows structure were conducted, respectively. Each test was repeated three times on the same bellows structure for repeatability validation. The resulting data of the repeated tests were compared with the analytical model in Figure 10, with the force predicted by the analytical model smaller than experimental measurements. This was probably due to the use of smaller stiffness values based on Taylor approximation. Nevertheless, both models proposed for single bellows structure and antagonistic bellows structure could capture the overall trend of the experiment results and effectively predict the axial force and displacement characteristics of the bellows structure. Particularly, the experimental data of a single bellows structure exhibited repeatable nonlinear pattern, whereas the antagonistic bellows structure showed improved linearity between the axial force and displacement. Least square curve fitting was conducted on the test data of antagonistic bellows structure in the working stroke interval with ${\hat{k}}_{a}=2.3167N∕mm$ and *R* > 0.99, validating the approximately linear stiffness by the proposed antagonistic design.

### Calibration for actuator displacement sensing

**FIG. 11.**
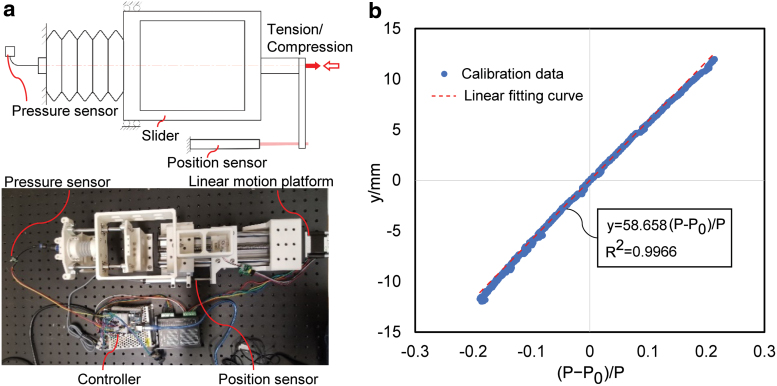
Calibration experiment of actuator displacement sensing. **(a)** Experimental setup. **(b)** Calibration data and results. The slope of the *red dotted curve* identifies the unknown parameter *S*. Color images are available online.

A key step of the proposed mechanoreception scheme was to estimate actuator displacement using the measured air pressure in the actuator chambers. However, this required the initial volume and initial pressure of the bellows chamber. Although the latter could be measured by the pressure sensor, the former had to be determined through calibration. A calibration experiment was conducted to identify the parameter of the displacement estimation model. As shown in Figure 11a, the same test apparatus was employed to apply the tensile or compressive deformation on the sealed bellows chamber connected with a pressure sensor (XGZP6847300KPGPN, range: −100 to 300 kPa; CFSENSOR Ltd.). The actual displacement data was captured by the linear position sensor (KPM16, 75 mm max.; MIRAN Ltd.) and the corresponding pressure was recorded by the pressure sensor. The experiment was repeated three times for reliability.

According to the proposed estimation model for displacement from pressure,

$$y=S\left(P-P_{0}\right)∕P.$$

To calibrate the parameter *S*, the pressure data *P* can be transformed to the ratio of the pressure change with respect to the initial pressure and the current pressure ρ:

$$\rho =\left(P-P_{0}\right)∕P,$$

where *P*_0_ is the initial pressure of the bellows chamber.

The results of the calibration experiment are presented in Figure 11b, with the actual displacement against the ratio ρ exhibiting very good linearity (*R* > 0.996) and the parameter *S* estimated as Ŝ=58.658.

**FIG. 12.**
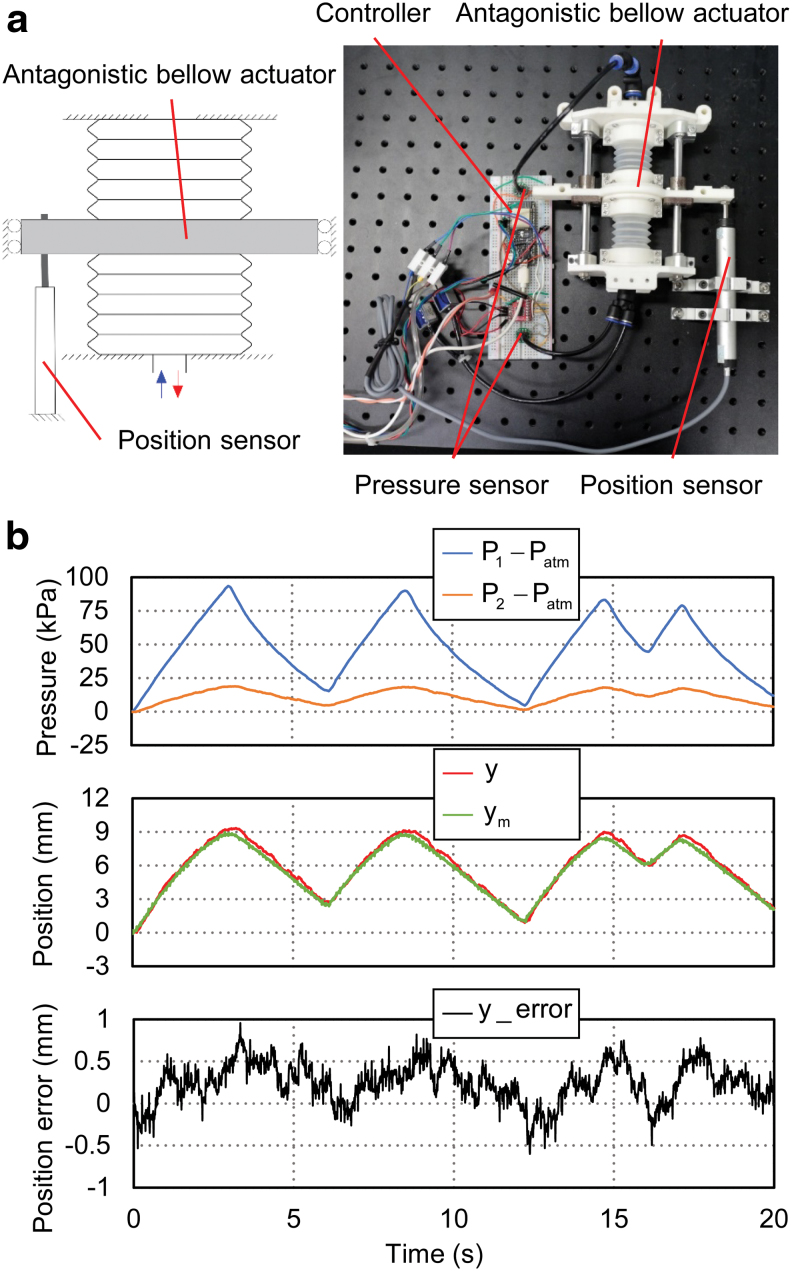
Validation experiment of actuator movement prediction. **(a)** Experimental setup. **(b)** Experimental results. *Top plots*: pressure of active bellow chamber *P*_1_ and passive bellow chamber *P*_2_ measured by the pressure sensors; *middle plots*: estimated displacement of actuator *y* and measured position *y_m_*; *bottom plots*: error *y*_error (*y* – *y_m_*) in actuator displacement estimation. Color images are available online.

To validate the calibration results, further experiments were carried out for actuator movement prediction, with results presented in Figure 12. In this experiment, movements of the antagonistic actuator were generated arbitrarily across the entire working range by inflating and deflating the active bellows chamber. The pressures of the two bellows chambers (*P*_1_ and *P*_2_) were tracked by the pressure sensors. Displacement of the actuator (*y_m_*) was measured by a linear position sensor and compared with the estimated value of the actuator movement (*y*) from the calibrated model. From the result shown in Figure 12b, the maximum absolute error between the measured and estimated displacement was 0.95 mm, with a mean of 0.238 mm and a standard deviation of 0.236 mm. The accuracy was sufficient in grasping tasks of general daily objects and contributed critically to the estimation of external contact forces.

### Experimental evaluation of external force estimation model

**FIG. 13.**
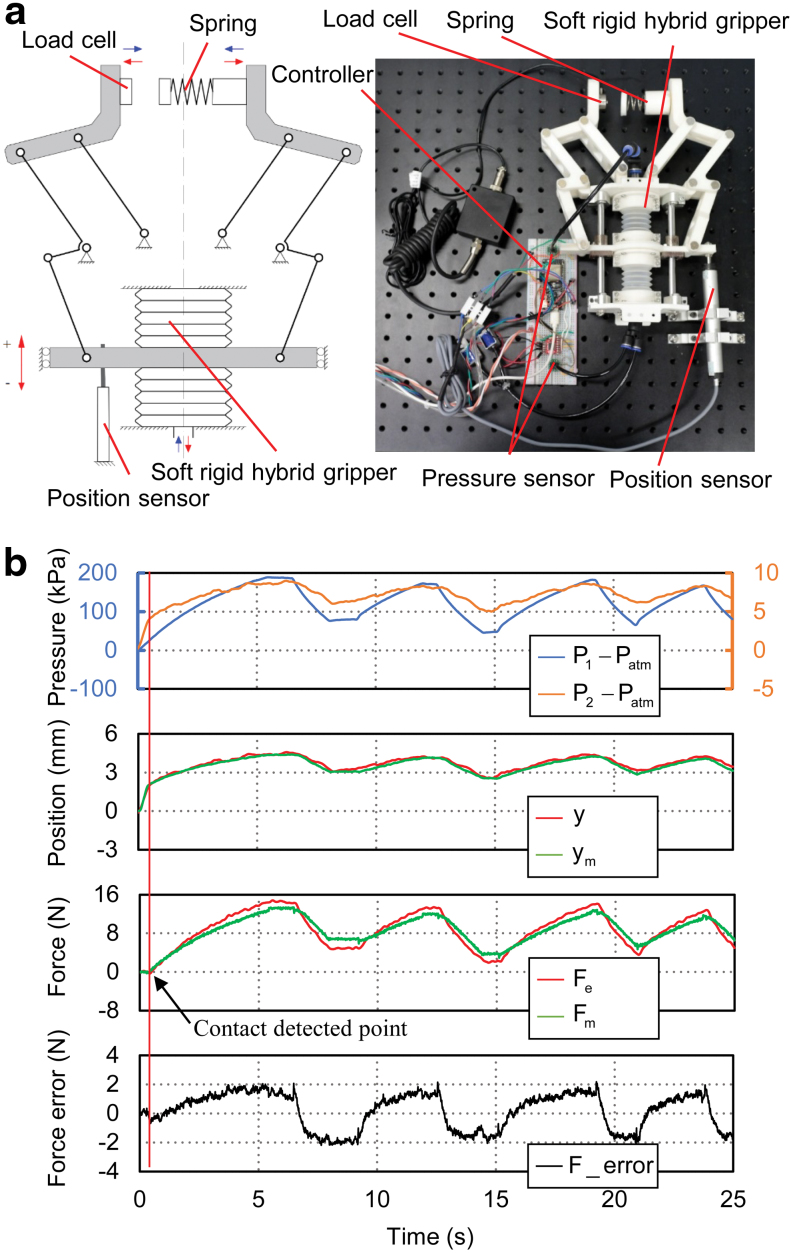
Validation experiment of external force estimation model. **(a)** Experimental setup. **(b)** Experimental results. From the *top*: pressure of active bellow chamber *P*_1_ and passive bellow chamber *P*_2_ measured by the pressure sensors; estimated displacement of actuator *y* and measured position *y_m_*; estimated external force *F_e_* and measured contact force *F_m_*; error *F*_error (*F_e_* − *F_m_*) in external force estimation. Color images are available online.

The proposed model for external contact force estimation was validated in an experiment when actuated movements and external mechanical stimuli were applied simultaneously. In the experiment, as illustrated in Figure 13a, the soft gripper was actuated to pinch a standard elastic object (a spring) by applying arbitrary gripping forces. A load cell was mounted onto one finger to record the contact force with the spring attached to the opposite finger.

The experimental results are presented in Figure 13b, showing the inner pressure of the active (*P*_1_) and passive (*P*_2_) bellows chambers, the estimated position of the actuator *y* and measured position *y_m_*, the actual contact force *F_m_* measured by the load cell and the estimated contact force *F_e_* from the derived models. The maximum error between the momentum observer estimation and the measured external force was 2.181 N, with a mean of 0.189 N and a standard deviation of 1.233 N. Some working-range correlated trends could be observed in the estimation error, which was possibly due to the model reduction and actuator hysteresis. In general, the estimation model performed well for the targeted purpose of obtaining the interactive force without requiring a dedicated force sensor. With the validated estimation accuracy, in future experiments, a reference force sensor was not used, as it would significantly interfere with the object grasping operations.

## Mechanoreception Experiments

**FIG. 14.**
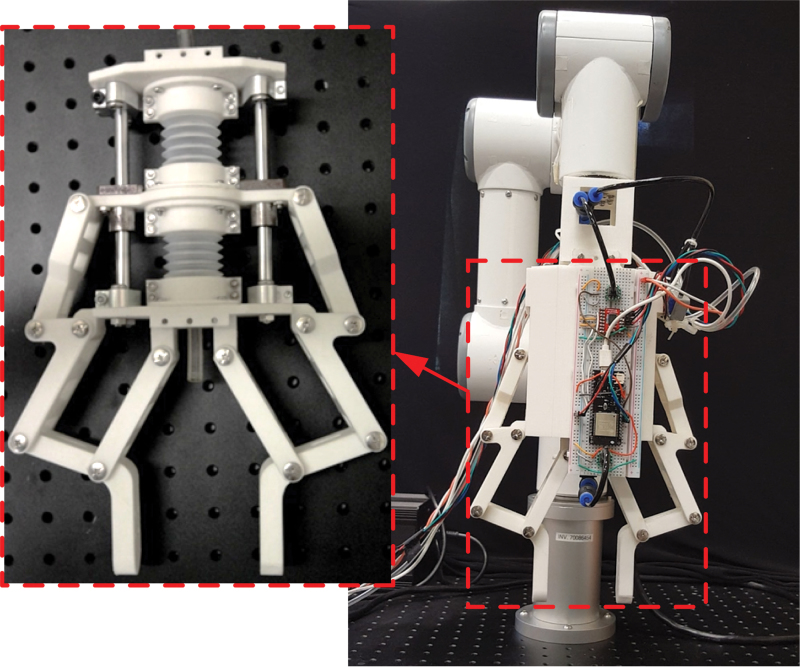
Prototype of the soft-rigid hybrid gripper with simple control system. Color images are available online.

In the final stage of validation, the prototyped soft-rigid hybrid gripper integrated with the antagonistic bellows actuator was tested by a series of object grasping experiments. A pneumatic control system was set up to drive the gripper with an air supply and two solenoid valves, one for inflation and the other for deflation. Two pressure sensors were connected to the bellows chambers to provide pressure feedback to the controller to determine the corresponding on/off states of the two solenoid valves, thus controlling the two fingers of the gripper. To fully demonstrate the mechanoreception capability of the gripper system in a practical setting, the gripper was mounted to a 6-DoF robotic manipulator arm (E6; SANTIFICO Ltd.) as shown in Figure 14.

In this section, to showcase the different features of the proposed mechanoreceptive gripper under different forms of mechanical stimuli, two kinds of interactive contacts are covered: active exploratory interaction and passive contact.

### Active exploratory interaction

**FIG. 15.**
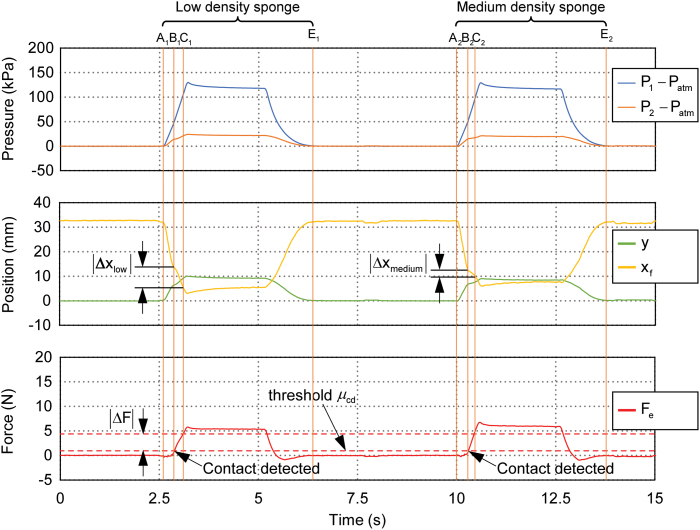
Stiffness identification of low-/medium-density sponges by single grasping. *Top plots*: pressure of the active *P*_1_ and passive *P*_2_ bellow chambers; *middle plots*: estimated actuator displacement *y* and right fingertip position *x_f_*; *bottom plots*: the estimated external contact force *F_e_*. Color images are available online.

With mechanoreception, the proposed gripper was capable of both contact detection and external contact force estimation. To highlight this capability, we demonstrated the gripper with a highly challenging task of object stiffness identification through a single grasping action. To illustrate the principle and implementation of object stiffness identification, the experimental results of grasping a low-density sponge (phase $A_{1}E_{1}$) and a medium-density sponge (phase $A_{2}E_{2}$) are shown in Figure 15. In these two grasping events, we focused on the phase from contact detection point *B_i_* to point *C_i_*, where the change of external contact force was ΔF relative to the contact detection point *B_i_* (i.e., threshold $\mu_{cd}$). Considering finger positions and the estimated external contact forces in the phase $B_{i}C_{i}$, distinctions can be readily observed for the two different density sponges: the low-density sponge suffered larger compressive deformation compared with the medium-density sponge. The ratio of the grasping force ΔF and the corresponding compressive deformation Δx in phase $B_{i}C_{i}$ (i.e., $\frac{\Delta F}{\Delta x_{low}}$ for low-density sponge and $\frac{\Delta F}{\Delta x_{medium}}$ for medium-density sponge) provided an estimation of the stiffness for distinguishing the two different sponges.

**FIG. 16.**
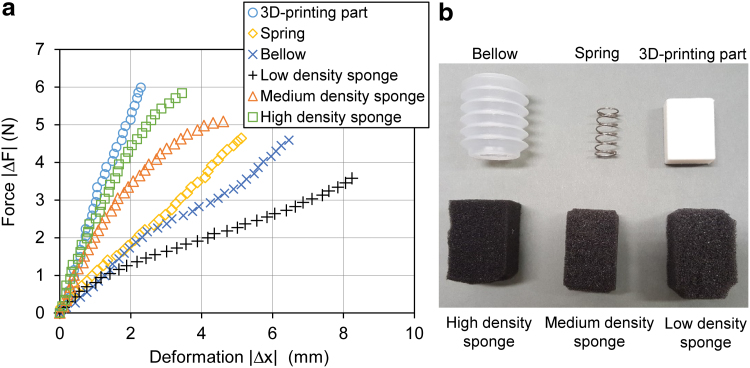
Force and deformation characteristics of six different objects explored by the gripper system by single grasping. **(a)** Plots of force and deformation data of each object captured by the gripper system. **(b)** Six objects characterized in the experiment. Color images are available online.

A group of six different objects, each with distinctive deformation properties, were used to further showcase the haptic sensing capability of the proposed gripper based on the same principle discussed earlier: (1) a rigid three-dimensional-printed cube (PLA material), (2) a spring (304# steel), (3) a bellows chamber (LDPE), (4) a low-density sponge, (5) a medium-density sponge, and (6) a high-density sponge. As shown in Figure 16a, the force-deformation scatter plot of the six objects were generated by recording the relative change of fingertip position and estimated contact force relative to the contact point. In general, the gripper system effectively painted the stiffness characteristics of the objects by a single grasping action without either a dedicated position sensor or force sensor.

### Passive mechanical stimuli detection

**FIG. 17.**
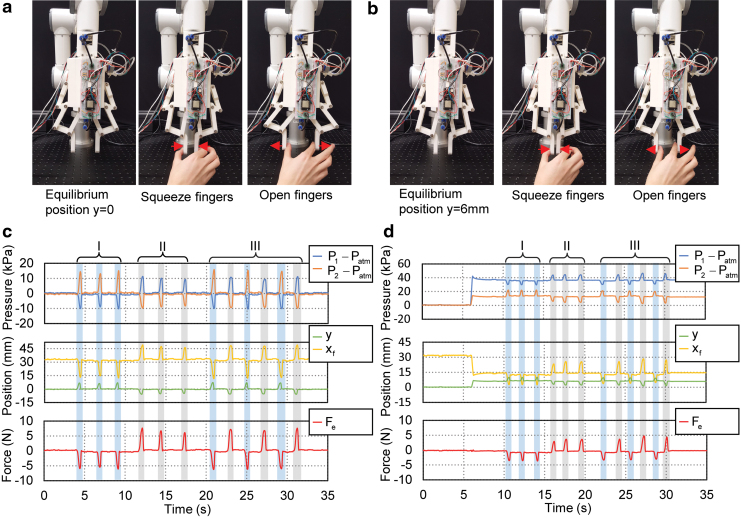
Experimental behavior of the gripper system when the two fingers being passively squeezed and opened. **(a)** Squeeze or open the two fingers when the actuator at equilibrium position *y* = 0. **(b)** Squeeze or open the two fingers when the actuator at equilibrium position *y* = 6 mm. **(c)** Experimental data for the gripper undergoing a sequence of open and squeezing at equilibrium position *y* = 0. **(d)** Experimental data for the gripper undergoing a sequence of open and squeezing at equilibrium position *y* = 6 mm. For **(b, d)**, *top plots*: pressure of the active *P*_1_ and passive *P*_2_ bellow chambers; *middle plots*: estimated actuator displacement *y* and right fingertip position *x_f_*; *bottom plots*: estimated external contact force *F_e_*. Color images are available online.

The scenario that the gripper was under the equilibrium state without active movements had also been considered. In this case, both the two bellows chambers of the actuator were sealed and the gripper system passively received the mechanical stimuli from the environment. In the first experiment, as shown in Figure 17, the two fingers were squeezed or force-opened by hand, while the changes of pressures in the two bellows chamber, the estimated position, and external contact force were recorded. During squeezing the active bellows chamber was elongated, whereas the passive bellows chamber was compressed. Hence, pressure in the active bellows chamber decreased, whereas the passive chamber pressure increased, that is, $\Delta P_{1}<0$ and $\Delta P_{2}>0$. We could also observe corresponding changes in the estimated positions and external contact forces, that is, Δy>0, $\Delta x_{f}<0$, and $\Delta F_{e}<0$. During the opening of the two fingers, opposite responses could be observed. Therefore, the proposed actuator could also act as a compound sensor of synchronous measurement of displacement and force, which could be explored from two practical aspects with passive mechanical stimuli detection: detecting contract loss and object contour scanning.

**FIG. 18.**
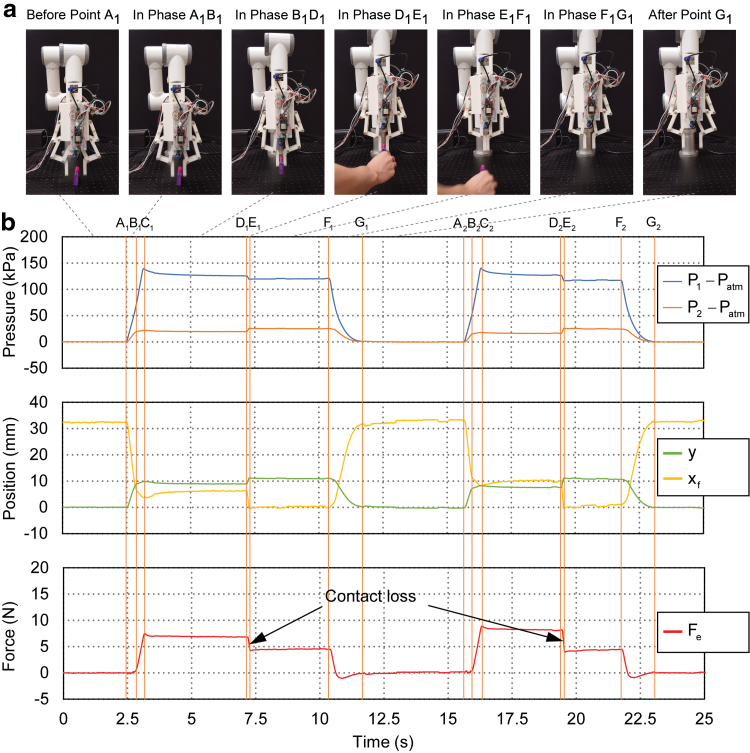
Experimental behavior of contact loss detection in object grasping. **(a)** Photographs during interaction sequence of the first grasping and contact loss event. Contact loss happened at Point *D*_1_ when the object tugged out from the two fingers. **(b)** Experimental data for the two grasping and contact loss event. *Top plots*: pressure of the active *P*_1_ and passive *P*_2_ bellow chambers; *middle plots*: estimated actuator displacement *y* and right fingertip position *x_f_*; *bottom plots*: estimated external contact force *F_e_*. Color images are available online.

A straightforward application was contact loss detection in object grasping. This provided the robot control system binary feedback that whether the object was successfully grasped or dropped from the fingers due to slippage, disturbances from robot arm movements, or other causes. We conducted two preliminary object grasping experiments during which contact losses were induced by tugging out manually the object held by the gripper fingers. As shown in Figure 18, the gripper was inflated at a pressure of 230 KPa to ensure successful grasping of the object in phase $A_{i}C_{i}$ and kept at the equilibrium state before point *D_i_*. The object was manually tugged out from the two fingers at point *D_i_*. In phase $D_{i}E_{i}$, the two fingers lost contact with the object and rapidly closed in with each other, reaching the new equilibrium state at point *E_i_*. As anticipated, we could observe a sudden decrease of pressure in the active bellows chamber, position of the fingers, and the external contact force, whereas the pressure of the passive bellows chamber and the corresponding position of the actuator underwent a jump in phase $D_{i}E_{i}$. A threshold $\mu_{cld}$ could be introduced to signal contact loss detection as$cld\left[F_{e}\left(t\right)\right]$ with ${Ḟ}_{e}<\mu_{cld}$.

**FIG. 19.**
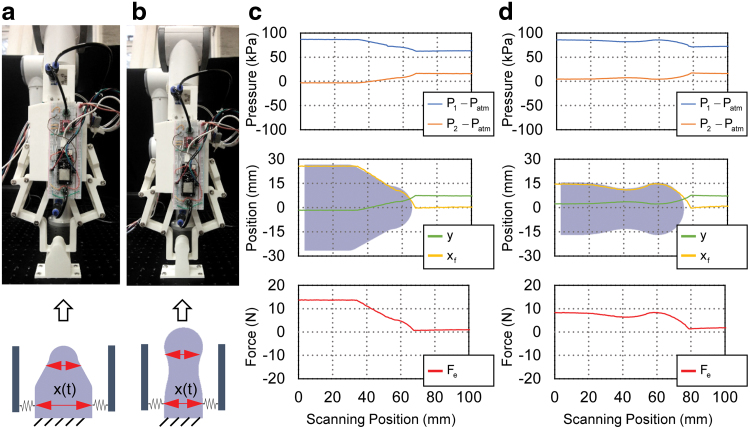
Object contour scanning experiments. **(a)** Experimental setup and schematics for contour scanning of a symmetrical tapered surface. **(b)** Experimental setup and schematics for contour scanning of a symmetrical waved surface. **(c)** Contour scanning results of the symmetrical tapered surface. **(d)** Contour scanning results of the symmetrical waved surface. For **(c, d)**, *top plots*: pressure of the active *P*_1_ and passive *P*_2_ bellow chambers; *middle plots*: estimated actuator displacement *y* and right fingertip position *x_f_*; *bottom plots*: estimated external contact force *F_e_*. Color images are available online.

Another interesting application was object contour scanning. In this application, the gripper was equipped with two new fingers designed with rounded tips. The robot arm was used to guide the gripper to scan a symmetrical tapered surface and a symmetrical waved pattern along *Z*-axis as shown in Figure 19a and c. Starting by clamping one end of the surface with enough gripper force, the two rounded fingertips were passively opened or closed with guided movement along *Z*-axis, conforming to the scanned surface. By recording the fingertip position *x_f_*, the contour of the surface can be traced. The experimental data of the two tested surfaces are shown in Figure 19b and d, respectively, exhibiting good matching with the real contour of the measured objects.

It is worth mentioning that the aforementioned experiments demonstrating passive mechanical stimuli detection showcased the advantages and potential of the inherent compliance and adaptability of soft robots. In particular, in object contour scanning, the passive compliance of the proposed elastic actuator was cunningly leveraged. In addition, an inherently compliant and adaptive actuator endowed with mechanoreception is highly promising in haptic exploration and dexterous manipulation tasks.

## Conclusions and Future Study

In this study, we provided a new scheme to endow soft pneumatic robots with proprioceptive sense in signaling body movement and the capability of exteroceptive contact detection. The concept was implemented and validated on a soft-rigid hybrid gripper system driven by a 1-DoF pneumatic bellows actuator. We demonstrated theoretically and experimentally that both actuator displacement and external contact force can be decoded simultaneously based on static and dynamic models and the pressure measurements of the bellows chambers. Notably, it was demonstrated that the proposed soft-rigid hybrid gripper endowed with mechanoreception could obtain quantitative understandings of the external environment (e.g., object stiffness and contour) without requiring dedicated sensors.

It is worth noting that the sealed chamber, in addition to the active chamber, was the key to providing a reference that enabled higher-level position and force estimations. The gas pressure sensors, on which the proposed mechanoreception scheme was based, were typically available in most soft pneumatic robots with deformable chambers for basic control. In light of this, the proposed mechanosensory scheme can be generalized to those soft extending and contracting actuators and can be of reference significance for the future development of smart soft robots.

There are two aspects of limitations regarding the proposed approach that can be addressed in future research. The working range was limited to the linearized stroke interval of the proposed antagonistic actuator, which restrained the overall performance of the gripper system. In future study, remedies to release this constraint will be sought to increase the working stroke in spite of the nonlinearity. Owing to the performance limitations of the soft actuator, this study was focused on 1-DoF linear motion. We will make efforts on the improvement of soft actuator design and extending the mechanosensory exploration to multi-DoF systems in future study. Both directions can substantially contribute to the generalizability of the proposed mechanoreception scheme for a wider range of soft robotic designs and systems.

## Appendix 1

### Dynamic Modeling of the Gripper System

#### Kinematic analysis

In o*xy* plane, the Cartesian coordinates of the mass center of the links are dependent on each other through the following equations:

(A1) $$x_{C_{1}}=0,y_{C_{1}}=y.$$

(A2) xC2=xC+rc2 sinα,yC2=yC1+rc2 cosα.

(A3) xC3=xC+rc3 sinθ−θ0+β0,yC3=yC+rc3 cosθ−θ0+β0.

(A4) xC4=xE+rc4 sinθ−θ0,yC4=yE+rc4 cosθ−θ0.

(A5) $$x_{C_{5}}=x_{C}+l\sin\left(\theta -\theta_{0}\right)-s,y_{C_{5}}=y_{C}+l\cos\left(\theta -\theta_{0}\right)+h.$$

The closed-loop 1 satisfies the following two constrained equations:

(A6) Lsinα=Rsinθ,

(A7) $$y+L\cos\alpha -y_{C}=R\cos\theta .$$

Differentiating the two constraints in Equations (A6) and (A7) with respect to time, we have

(A8) $$L\dot{\alpha }\cos\alpha =R\dot{\theta }\cos\theta ,$$

(A9) $$ẏ-L\dot{\alpha }\sin\alpha =-R\dot{\theta }\sin\theta .$$

Consider that the gripper system is one degree of freedom, displacement *y*, angle α, and their derivatives can be rewritten in terms of θ and $\dot{\theta }$ by applying the relation from Equations (A8) to (A9)

(A10) $$\dot{\alpha }=\frac{R\cos\theta }{c}\dot{\theta },$$

(A11) ẏ=R2 sinθcosθc−Rsinθθ˙,

where $c=\sqrt{L^{2}-R^{2}{s in}^{2}\theta }$.

#### Dynamic model

Lagrange equation will be applied to derive the dynamic model of the system. We first determine the total kinetic energy and the total potential energy of the gripper system.

The kinetic energies of the links are, respectively,

(A12) T1=12m1ẏ2=12m1R2 sinθcosθc−Rsinθ2θ˙2,

(A14) $$T_{3}=\frac{1}{2}I_{3}{\dot{\theta }}^{2}+\frac{1}{2}m_{3}{ẋ}_{C3}^{2}+\frac{1}{2}m_{3}{ẏ}_{C3}^{2}=\frac{1}{2}\left(I_{3}+m_{3}r_{c3}^{2}\right){\dot{\theta }}^{2},$$

(A15) $$T_{4}=\frac{1}{2}I_{4}{\dot{\theta }}^{2}+\frac{1}{2}m_{4}{ẋ}_{C4}^{2}+\frac{1}{2}m_{4}{ẏ}_{C4}^{2}=\frac{1}{2}\left(I_{4}+m_{4}r_{c4}^{2}\right){\dot{\theta }}^{2},$$

(A16) $$T_{5}=\frac{1}{2}m_{5}{ẋ}_{C3}^{2}+\frac{1}{2}m_{5}{ẏ}_{C3}^{2}=\frac{1}{2}m_{5}l^{2}{\dot{\theta }}^{2}.$$

The total kinetic energy *T* of the gripper system can be expressed as

(A17) $$T=T_{1}+2\sum_{i=2}^{5}T_{i}=J\left(\theta \right){\dot{\theta }}^{2},$$

where

The potential energies of the links due to gravity are, respectively,

(A18) $$U_{g,1}=m_{1}gy_{c1}=m_{1}g\left(R\cos\theta +y_{C}-c\right),$$

(A19) $$U_{g,2}=m_{2}gy_{c2}=m_{2}g\left[R\cos\theta +y_{C}-c\left(1-\frac{r_{c2}}{L}\right)\right],$$

(A20) Ug,3=m3gyc3=m3gyC+rc3 cosθ−θ0+β0,

(A21) Ug,4=m4gyE+rc4 cosθ−θ0,

(A22) $$U_{g,5}=m_{5}g\left[y_{C}+l\cos\left(\theta -\theta_{0}\right)+h\right].$$

Assume that

(1) The base coordinate is $\left\{U\right\}$ and the gripper coordinate is $\left\{G\right\}$.
(2) The direction of gravity is $-Z_{U}$.

The gravitational potential energy $G\left(\theta \right)$ of the gripper system can be determined as

(A23) $$G\left(\theta \right)=\left(-{\hat{Y}}_{G}\cdot Z_{U}\right)\left(U_{g,1}+2\sum_{i=2}^{5}U_{g,i}\right).$$

Because the antagonistic bellows actuator performs with approximately linear stiffness in the work stroke, the elastic potential energy$U\left(\theta \right)$ stored in the antagonistic bellows structure can be formulated as

(A24) $$U\left(\theta \right)=\frac{1}{2}k_{a}y^{2}=\frac{1}{2}k_{a}{\left(R\cos\theta +y_{C}-c\right)}^{2}.$$

Thus, the total potential energy *V* of the gripper system can be expressed as

The Lagrange function *L* is determined as

(A26) $$L\equiv T-V=J\left(\theta \right){\dot{\theta }}^{2}-G\left(\theta \right)-U\left(\theta \right).$$

The virtual work done by the actuation force with virtual displacement δy and external force applied on the gripper finger along *x*-axis with the virtual displacement δx is given by

(A27) δW=Faδy+Feδx=Fa∂y(θ)∂θ+Fe∂x(θ)∂θδθ=FaR2 sinθcosθc−Rsinθ+Felcosθ−θ0δθ,

where$F_{a}=\frac{1}{4}\pi d^{2}\left(P_{1}-P_{2}\right)$.

Applying the Lagrange's equation in the following form:

(A28) $$\frac{ud}{dt}\left(\frac{\partial L}{\partial \dot{\theta }}\right)-\frac{\partial L}{\partial \theta }=Q_{\theta },$$

where$Q_{\theta }=\frac{\delta W}{\delta \theta }=\tau_{a}\left(P_{1},P_{2},\theta \right)+\tau_{e}\left(\theta \right)=F_{a}\frac{\partial y\left(\theta \right)}{\partial \theta }+F_{e}\frac{\partial x\left(\theta \right)}{\partial \theta }$.

Substituting Equation (A26) into Equation (A28), we can summarize the dynamic model of the system in the following form:

(A29) $$M\left(\theta \right)\ddot{\theta }+C\left(\theta ,\dot{\theta }\right)\dot{\theta }+g\left(\theta \right)+u\left(\theta \right)=Q_{\theta },$$

where
